# Anthropogenic Vessel Strike as a Threat to Spotted Seals (*Phoca largha*) in Korean Waters: A Multimodal Forensic Investigation

**DOI:** 10.3390/ani16091306

**Published:** 2026-04-23

**Authors:** Ji-Hyung Park, Hae Suk Choi, Daji Noh, Sooyoung Choi, Seung Hyeok Seok, Sang Wha Kim, Adams Hei Long Yuen

**Affiliations:** 1 College of Veterinary Medicine & Institute of Veterinary Science, Kangwon National University, Chuncheon 24341, Republic of Korea; mung90@kangwon.ac.kr (J.-H.P.); djnoh@kangwon.ac.kr (D.N.); choisooyoung@kangwon.ac.kr (S.C.); 2 Department of Microbiology and Immunology, Institute of Endemic Disease, Seoul National University College of Medicine, Seoul 03080, Republic of Korea; csh6512@naver.com (H.S.C.); lamseok@snu.ac.kr (S.H.S.); 3 School of Medical and Health Sciences, Tung Wah College, Homantin, Kowloon, Hong Kong SAR, China

**Keywords:** spotted seal, marine mammal forensics, post-mortem computed tomography, drowning, propeller strike

## Abstract

The spotted seal (*Phoca largha*) is a flagship species and natural monument inhabiting Korean coastal waters. Due to its conservation importance and the rarity of carcass discoveries, determining the cause of death of each individual is critical. A juvenile female spotted seal carcass was discovered on the eastern coast of Korea in May 2025. External examination revealed multiple parallel lacerations consistent with propeller strike injuries. Post-mortem computed tomography (PMCT) was performed prior to necropsy to provide a comprehensive forensic analysis. CT imaging revealed the longest wound measured 10.49 cm in length and 1.58 cm in depth, suggesting a minimum propeller diameter of approximately 19 cm. Skeletal injuries included a coccygeal vertebral fracture and subluxation of the left astragalus and calcaneus. CT images of the respiratory tract showed frothy fluid in the nasal cavity and trachea, as well as ground-glass opacity and consolidation in the lung parenchyma. Necropsy findings confirmed severe pulmonary edema, congestion, and abundant frothy foam throughout the respiratory tract. Histological analysis revealed pulmonary edema with eosinophilic fluid and erythrocytes in alveolar spaces, markedly distended blood vessels, and intra-alveolar hemorrhage. This comprehensive approach demonstrated that the cause of death was drowning, secondary to propeller strike by a small vessel (<4.5 m). To the authors’ knowledge, this is the first case report providing a detailed forensic analysis of a juvenile spotted seal found on the eastern coast of Korea. This case highlights the importance of integrating PMCT with conventional necropsy to improve cause-of-death determination in marine mammal conservation.

## Simple Summary

Spotted seals are protected marine mammals in Korea, and understanding the cause of death of each individual is important for their conservation. In this study, we investigated a juvenile spotted seal found dead on the eastern coast of Korea. The animal had severe injuries caused by a boat propeller, which damaged its hindlimbs and made swimming impossible. To determine the cause of death, we applied a multidisciplinary approach combining post-mortem CT scans with conventional necropsy and microscopic examination. We found strong evidence of drowning, including fluid and foam in the airways and severe lung damage. These findings show that the seal likely died from drowning after being struck by a small vessel. Our results demonstrate that integrating imaging with traditional necropsy improves the accuracy of mortality investigations and helps identify human-related threats. This approach can provide valuable information for conservation strategies and contribute to better protection of vulnerable marine mammal populations in Korean waters.

## 1. Introduction

The spotted seal (*Phoca largha*) is a flagship marine mammal species in Korea, reflecting its ecological and conservation significance [1]. Spotted seals occurring in Korean coastal waters are broadly divided into two major population groups based on their geographic distribution and migratory behavior. The Yellow Sea population primarily breeds in Liaodong Bay, China, and utilizes the Tae-An Peninsula in South Korea as a resting site, forming a relatively closed-loop migration pattern as an isolated population [2]. Notably, a significant aggregation of this population is observed around Baengnyeong Island, South Korea, where recent studies have reported a declining population trend [3]. In contrast, the East Sea (Sea of Japan) population breeds in Peter the Great Bay, Russia, and generally migrates northward, although some individuals occasionally venture beyond their range southward to Korean waters [4,5].

Despite being classified as “Least Concern” on the IUCN Red List (IUCN) [6], spotted seals are increasingly exposed to anthropogenic pressures, including commercial fisheries and intensive marine activities, which threaten local populations [5,7]. These pressures raise concerns about potential regional declines. Accordingly, the species is legally protected in Korea as both a Natural Monument and a Class I endangered wildlife species [8,9]. Given these conservation concerns, each mortality event involving a spotted seal in Korean waters provides a valuable opportunity to investigate the cause of death and generate critical insights into marine ecosystem health and species conservation.

Conventional necropsy has long served as the mainstay of marine mammal stranding investigations, enabling gross pathological assessment and sample collection. However, conventional necropsy alone may be insufficient for detecting subtle internal trauma, characterizing complex injury patterns, or providing objective documentation of findings prior to tissue disruption during dissection. In recent years, postmortem computed tomography (PMCT) has emerged as a routine complementary modality for the examination of deceased marine mammals in Korean waters [10,11,12], offering non-invasive, electronic documentation of skeletal and soft tissue structures that can guide subsequent necropsy and enhance diagnostic accuracy. The electronic documentation also serves as a permanent, objective record that can be revisited for secondary analysis, peer review, or retrospective biodiversity studies—a critical advantage for long-term marine mammal conservation research [13].

The present study describes the investigation of a spotted seal recovered from the Eastern Coast of Korea. The specimen underwent whole-body PMCT prior to necropsy and histopathological analysis. The findings illustrate the diagnostic utility of integrating imaging with conventional examination methods in marine mammal stranding investigations and highlight the forensic characteristics of propeller strike injury—an anthropogenic trauma of growing concern amid increasing vessel traffic in Korean coastal waters.

## 2. Materials and Methods

### 2.1. Carcass Recovery

**Figure 1:**
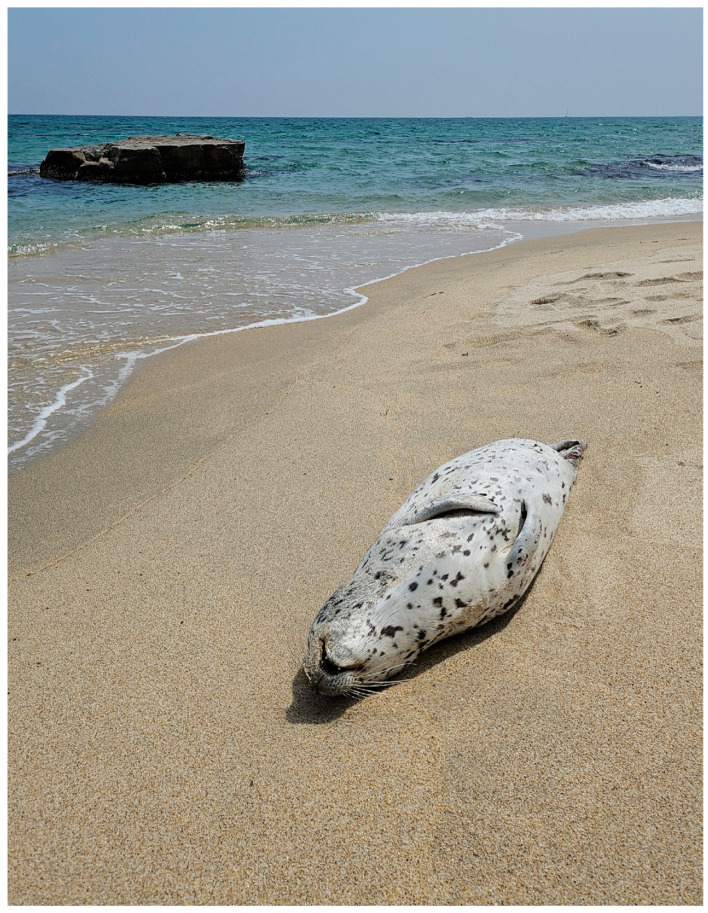
Carcass of a juvenile spotted seal found on Gangneung Beach, eastern coast of Korea.

A carcass of a female spotted seal was discovered on Gangneung beach (37.706783° N, 129.014857° E) on the eastern coast of Korea in early May 2025 (Figure 1). The nearest known breeding ground to the discovery location is Peter the Great Bay, where parturition typically occurs between February and March [14]. Considering the timing of the breeding season and the small size of the carcass, the discovered specimen was estimated to be a juvenile of less than six months of age. The time of death was reasonably inferred based on eyewitness observation of the animal exhibiting labored breathing and taking its final breaths prior to death. The carcass was retrieved the following day. Upon movement, malodorous watery brown fluid was observed leaking from the oral cavity.

### 2.2. PMCT Scanning

PMCT scanning was performed as a pre-necropsy screening procedure within approximately 48 h postmortem using a 16-slice CT scanner (Alexion™, Canon Medical Systems, Otawara, Japan). The carcass was scanned using 100 kVp, 250 mAs, with a section thickness of 1.0 mm. The field of view was set at 350 mm to encompass the entire body girth. Sequential transaxial data were post-processed to generate three-dimensional volume-rendered images using a commercial image post-processing system (Synapse 3D, Fujifilm, Tokyo, Japan). Image analysis included three-dimensional skeletal reconstruction and multiplanar reconstruction for detailed assessment of osseous and soft tissue structures.

### 2.3. Necropsy and Sample Collection

Systematic necropsy was performed following imaging examination, according to standard protocols for marine mammals [15]. External examination included measurement of body length and body weight, assessment of dentition, and documentation of external skin lesions. An internal examination was conducted to evaluate all major organs, and gross findings were recorded. Tissue samples from the lung, liver, spleen, and kidney were collected and fixed in 10% neutral buffered formalin for histological examination. The tissues were processed using a paraffin-embedded workflow, followed by sectioning and hematoxylin and eosin (H&E) staining. Parasites recovered from the stomach were stored at −20 °C for subsequent PCR analysis.

## 3. Results

### 3.1. External Examination

**Figure 2:**
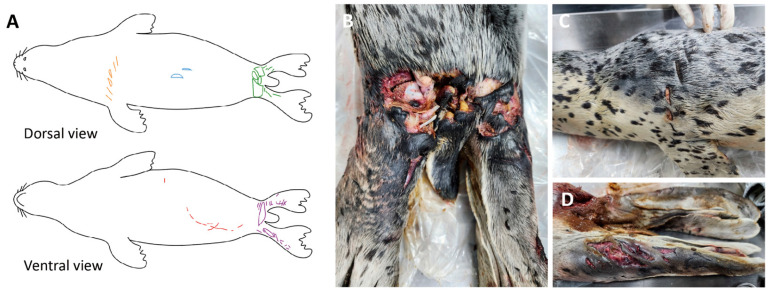
External propeller-induced skin injuries in a spotted seal. (**A**) Schematic representation of skin wounds. The body surface was divided into five regions and color-coded according to location: (1) left scapular region (yellow), (2) middle dorsum (sky blue), (3) ventrum (red), (4) dorsal aspect of the hindlimb (green), and (5) ventral aspect of the hindlimb (purple). (**B**) Severe injury to the dorsal caudal body region, with exposed muscle, bone and transected tendons. (**C**) Parallel linear lacerations on the left scapular region. (**D**) Multiple parallel linear wounds on the lateral aspect of the hindlimb.

Morphometric measurements included: body weight 22.4 kg, total length 114.3 cm, and nose-to-tail length 100 cm. Dental examination confirmed the presence of all permanent teeth (incisors 3, canine 1, postcanines 5). Body condition scoring indicated a robust condition, with blubber thickness measuring 2.6 cm dorsally, 2.8 cm laterally, and 2.5 cm ventrally around the umbilical region. As the carcass was relatively fresh, having been deceased for slightly over 24 h, it was classified as decomposition code 2 [15]. External examination revealed multiple traumatic injuries distributed across five anatomical regions: (1) left scapular region, (2) middle dorsum, (3) ventrum, (4) dorsal aspect of hindlimb, and (5) ventral aspect of hindlimb (Figure 2A). Wounds penetrating deeper than the dermis contained sand particles. The left scapular region exhibited parallel beveled lacerations with approximately 1.7 cm spacing between scars. The most severe trauma was localized to the hindlimb region, particularly the dorsal gluteal area, which displayed transverse laceration with torn skin exposing underlying bone. Deep muscle lacerations were present, with complete rupture of three tendons in the left leg (Figure 2B–D).

### 3.2. PMCT Findings

**Figure 3:**
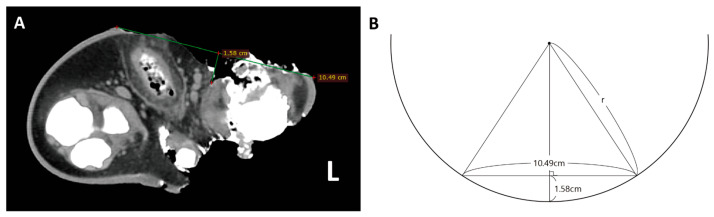
CT-based measurement of wound dimensions and propeller size estimation. (**A**) CT image demonstrating measurement of the longest diameter of a transverse tail wound (10.49 cm in length; 1.58 cm in depth). (**B**) Estimation of the propeller radius based on these measurements.

**Figure 4:**
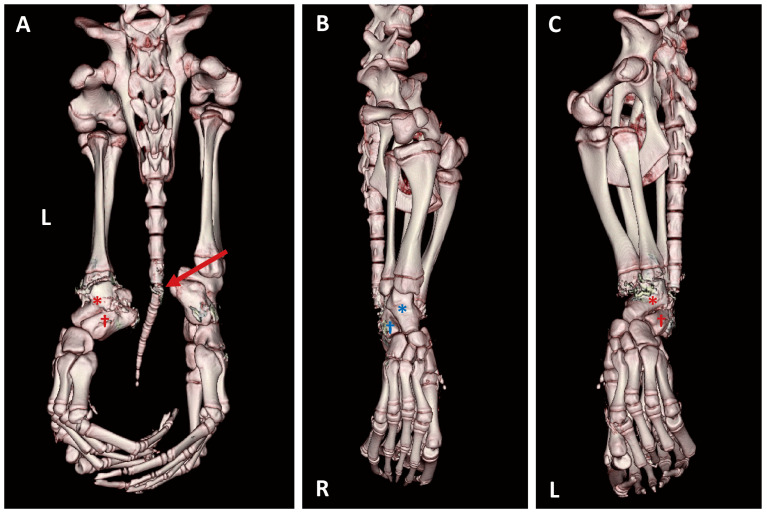
Three-dimensional reconstructed CT images of skeletal injuries. Asterisk (*) indicates the astragalus; dagger (†) indicates the calcaneus. Abnormal findings are indicated in red, whereas the contralateral normal structures are indicated in blue for comparison. Sand artifacts are present around the affected regions. (**A**) Dorsoventral view showing a distinct coccygeal vertebral fracture (arrow) and subluxation of the astragalus (*) and calcaneus (†). (**B**) Right lateral view demonstrating normal alignment of the right astragalus (*) and calcaneus (†). (**C**) Left lateral view showing subluxation of the astragalus (*) and calcaneus (†) with dorsomedial displacement compared to the contralateral side.

**Figure 5:**
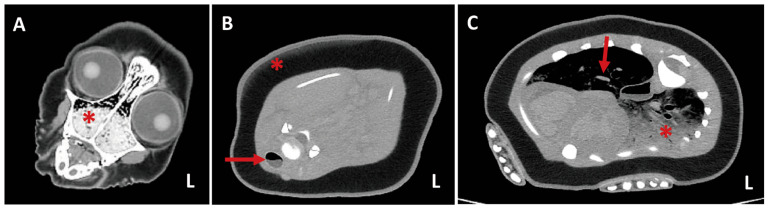
Transaxial CT findings of the cranial body of a spotted seal. (**A**) Head region showing frothy fluid within the nasal cavity (asterisk). (**B**) Section at the scapular level demonstrating fluid within the tracheal lumen (arrow) and superficial subcutaneous blubber contusion in the right periscapular region (asterisk). (**C**) Ground-glass opacity in the left lung (asterisk) with intrabronchial fluid accumulation (arrow). Dependent changes possibly related to livor mortis in the left lung are also noted, likely influenced by a dorsoventral position with slight leftward tilt.

Cutaneous wound dimensions were measured using CT-based calipers. The longest lesion was located on the ventral aspect of the hindlimb, measuring 10.49 cm in length and 1.58 cm in depth, measuring 10.49 cm in length and 1.58 cm in depth, based on CT software measurements (Figure 3A). The minimum propeller diameter was calculated as approximately 19 cm (2r ≒ 19 cm) (Figure 3B) [16]. Three-dimensional skeletal reconstruction revealed a fracture between the 6th and 7th coccygeal vertebrae, subluxation of the left astragalus and calcaneus with medial displacement, and erosion of the cranial aspect of the left astragalus (Figure 4). The exposed bone visible through the torn skin was identified as the left astragalus. Multiple major tendons associated with the Achilles’ tendon complex were completely transected. Respiratory tract imaging demonstrated frothy fluid in the maxilloturbinate region and fluid accumulation within the trachea extending from the tracheal origin to the area near the humerus. Pulmonary findings included ground-glass opacity of lung parenchyma, consolidation, and intrabronchial fluid. Additional findings included superficial blubber contusion in the right peri-scapular region (Figure 5).

### 3.3. Internal Examination

**Figure 6:**
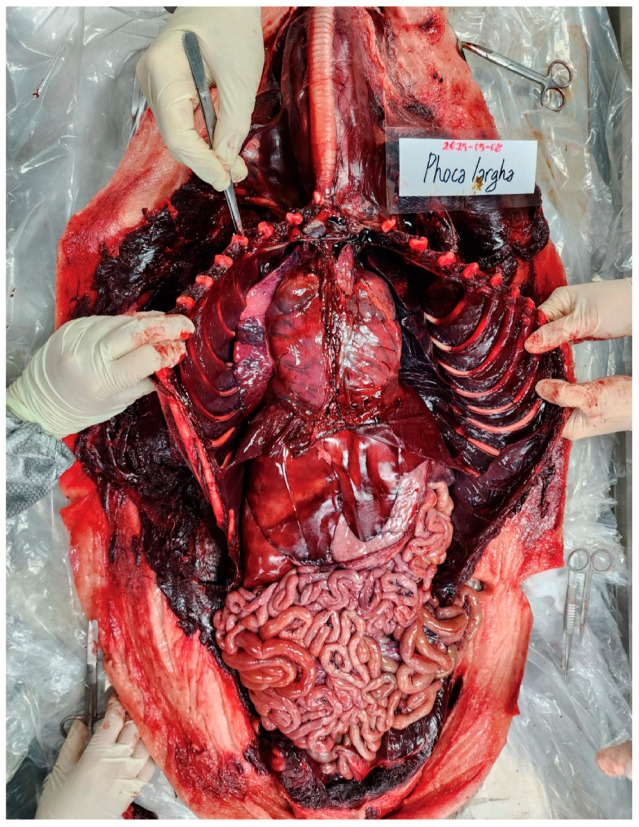
Macroscopic findings of the thoracic and abdominal cavities. Opened thoracic and abdominal cavities of the spotted seal, showing diffuse congestion of internal organs, including the heart, lungs, and liver. Skeletal muscles also exhibit diffuse dark red discoloration, consistent with systemic venous congestion.

The trachea contained abundant frothy foam extending from the distal trachea to the bronchi. Pulmonary examination revealed severe pulmonary edema and congestion, characterized by diffuse dark red discoloration (left > right), with whitish foam present within the bronchi. Gastric examination revealed no food contents but contained brown, malodorous fluid similar in appearance to the fluid observed leaking from the oral cavity. A total of 13 mL of fluid was collected from the stomach. Six nematodes recovered from the stomach were identified as *Pseudoterranova azarasi* by PCR analysis [17]. Intestinal examination revealed minimal to no luminal contents in the proximal intestine, whereas abundant fecal material was present in the distal portion. The liver exhibited mild rib impression and showed diffuse congestion, particularly in the caudal region (Figure 6).

### 3.4. Histopathological Analysis

Histological examination of the lung revealed severe, diffuse pulmonary edema and congestion, characterized by widespread alveolar spaces filled with eosinophilic fluid and erythrocytes (intra-alveolar hemorrhage), along with markedly distended blood vessels and numerous alveolar macrophages. The alveolar septa were diffusely congested and thickened by interstitial edema, and mild overinflation was also observed [18,19]. These pulmonary findings were the most prominent lesions and are consistent with acute respiratory distress associated with drowning, leading to terminal asphyxia.

**Figure 7:**
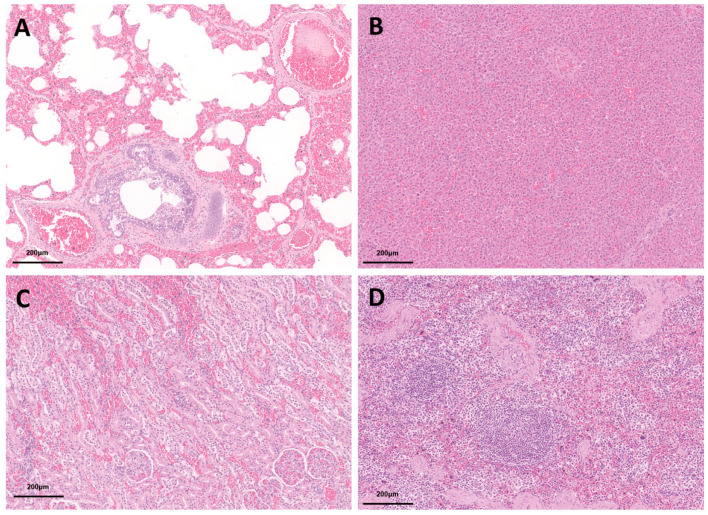
Histopathological findings (H&E stain). (**A**) Lung showing severe pulmonary edema and congestion with intra-alveolar hemorrhage. (**B**) Liver showing hepatocellular vacuolation and sinusoidal congestion. (**C**) Kidney showing diffuse congestion and mild tubular epithelial degeneration. (**D**) Spleen showing reactive lymphoid aggregates with red pulp congestion.

In comparison, extra-pulmonary organs showed changes consistent with systemic circulatory disturbance and hypoxia. Hepatic histopathology revealed diffuse hepatocellular vacuolation, characterized by multiple small cytoplasmic clear vacuoles with centrally located nuclei, along with sinusoidal congestion. Splenic histopathology showed reactive lymphoid aggregates within the white pulp and moderate congestion of the red pulp. Renal examination revealed diffuse congestion with marked engorgement of interstitial and peritubular capillaries. Mild to moderate tubular epithelial degeneration, including cellular swelling and cytoplasmic pallor, was observed, consistent with early hypoxic injury, while glomeruli were relatively preserved. No significant inflammatory or chronic lesions were identified (Figure 7).

## 4. Discussion

The pattern of injuries observed in this spotted seal is consistent with propeller strike by a small watercraft. Multiple parallel linear lacerations provide definitive evidence of propeller blade contact. Based on CT imaging analysis of the longest wound, the striking propeller had a minimum diameter of 19 cm, suggesting the involved vessel was a small outboard craft less than 4.5 m in length [20]. In Korean waters, such vessels typically include motor boats, yachts, and personal watercraft [21,22]. This case has underscored a broader conservation concern about the increasing overlap between recreational vessel operations and critical marine mammal habitats in Korea’s coastal waters. As coastal development and maritime tourism continue to expand, the risk of propeller strike injuries to protected marine species is likely to increase [23]. Documenting such incidents provides essential evidence to inform marine spatial planning, vessel speed restrictions, and the designation of protected areas—management tools that are increasingly recognized as vital for mitigating anthropogenic impacts on marine biodiversity.

The operational areas of small recreational vessels frequently overlap with habitats utilized by juvenile seals. Young seals, being in the process of adapting to aquatic locomotion, tend to frequent shallow waters and spend extended periods at the surface [24]. This behavior pattern has been documented through satellite tagging studies of underyearling individuals, which demonstrate coastal movement patterns [25]. Consequently, the probability of vessel-seal interactions increases when juvenile seals utilize shallow coastal waters frequented by small watercraft. Additionally, young seals lack extensive experience with vessel traffic and may be less adept at avoidance behaviors [23,26]. These behavioral vulnerabilities, combined with the increasing density of recreational vessels in coastal waters, elevate the conservation significance of each documented strike event.

The propeller strike inflicted severe damage to the seal’s caudal region and hindlimbs—anatomical structures critical for locomotory propulsion. Unlike sea lions, which utilize front flippers for propulsion, seals rely exclusively on hindlimb-generated thrust [27]. This specimen sustained bilateral hindlimb injuries affecting both dorsal and ventral aspects, with complete severance of the left hindlimb tendons. The coccygeal vertebrae were completely fractured. This condition effectively prevented thunniform undulation and rendered the seal unable to swim or maintain buoyancy, a functional consequence directly relevant to determining the cause of death.

While propeller strike injuries were clearly evident externally, death resulted from drowning secondary to these incapacitating injuries. The propeller strike inflicted severe damage to the caudal region and hindlimbs, which are critical for propulsion in seals. As a result, the animal would have been unable to perform effective swimming movements or maintain buoyancy, leading to submersion and subsequent drowning. The presence of frothy material within the upper respiratory tract, confirmed by both necropsy and CT imaging, provides strong evidence of perimortem aspiration. Additional findings, including diffuse congestion in multiple organs, further support systemic hypoxia due to oxygen deprivation. Histological examination of the lungs revealed severe pulmonary edema, congestion, and intra-alveolar hemorrhage, which represent the most prominent lesions and are consistent with drowning pathophysiology [18,19]. Although postmortem changes, including dependent livor mortis, may influence CT and histological findings, the relatively short postmortem interval suggests that such effects were likely limited. Extra-pulmonary findings—including hepatic sinusoidal congestion with hepatocellular vacuolation, renal congestion with mild tubular epithelial degeneration, and splenic congestion—are consistent with secondary systemic circulatory disturbance and acute hypoxic injury. No significant chronic lesions or underlying diseases were identified that could independently account for death. However, histopathological evaluation of cutaneous, musculoskeletal, and brain tissues was not performed, which may represent a limitation of the study.

In this case, the integration of PMCT with conventional necropsy and histopathological examination provided a more comprehensive evaluation of the cause of death. While gross and microscopic findings alone suggested drowning secondary to traumatic injury, PMCT offered additional objective evidence, including visualization of frothy material within the upper respiratory tract and detailed characterization of propeller-induced skeletal and soft tissue injuries. The use of PMCT allowed for non-invasive assessment of internal structures prior to dissection and facilitated more accurate correlation between traumatic lesions and functional impairment. This multimodal approach strengthened the diagnostic interpretation by linking severe locomotor disruption with subsequent drowning, thereby improving confidence in determining the cause of death. These findings highlight the value of PMCT as a complementary tool in marine mammal forensic investigations, particularly in cases involving traumatic injury where functional consequences are critical for interpreting mortality. Given that this study is based on a single case, individual variation should be considered.

Taken together, the combination of severe locomotor impairment due to propeller trauma and the characteristic pulmonary and systemic pathological findings strongly suggests that the animal died from drowning secondary to propeller strike injuries. However, as with most wildlife cases, definitive confirmation remains challenging and should be interpreted alongside environmental and circumstantial evidence.

## 5. Conclusions

To the authors’ knowledge, this is the first study in Korea to provide a detailed post-mortem analysis of a juvenile spotted seal using conventional necropsy complemented by PMCT. This study underscores the essential role of meticulous post-mortem examinations in identifying and understanding the threats facing spotted seals in Korean waters. The use of a multidisciplinary diagnostic approach enhanced our ability to investigate marine mammal mortality and provides a valuable framework for future forensic applications. Such comprehensive analyses not only strengthen the accuracy of diagnoses but also expand the scope of information available for assessing marine mammal health and mortality.

The findings from this investigation also contribute critical insights into anthropogenic impacts, including vessel strikes, and directly support the development of conservation and mitigation strategies. Given the designation of the spotted seal as a natural monument, systematic and continuous surveillance is indispensable for safeguarding the future of this vulnerable population.

## Data Availability

The original contributions presented in this study are included in the article. Further inquiries can be directed to the corresponding authors.
